# High Reflectivity and Thermal Conductivity Ag–Cu Multi-Material Structures Fabricated via Laser Powder Bed Fusion: Formation Mechanisms, Interfacial Characteristics, and Molten Pool Behavior

**DOI:** 10.3390/mi14020362

**Published:** 2023-01-31

**Authors:** Qiaoyu Chen, Yongbin Jing, Jie Yin, Zheng Li, Wei Xiong, Ping Gong, Lu Zhang, Simeng Li, Ruiqi Pan, Xiya Zhao, Liang Hao

**Affiliations:** 1 Gemmological Institute, China University of Geosciences, Wuhan 430074, China; 2 Hubei Gem & Jewelry Engineering Technology Research Center, Wuhan 430074, China; 3 School of Mechanical Engineering and Electronic Information, China University of Geosciences, Wuhan 430074, China; 4 Academy of Fine Arts, Guizhou Normal University, Guiyang 550001, China

**Keywords:** highly reflective and thermally conductive metals, multi-material structures, laser powder bed fusion, interfacial characteristics, Ag alloy, Cu alloy

## Abstract

Ag and Cu have different advantages and are widely used in key fields due to their typical highly electrical and thermal conductive (HETC) properties. Laser powder bed fusion (LPBF), an innovative technology for manufacturing metallic multi-material components with high accuracy, has expanded the application of Ag–Cu in emerging high-tech fields. In this study, the multi-material sandwich structures of Ag7.5Cu/Cu10Sn/Ag7.5Cu were printed using LPBF, and the formation mechanism, interface characteristics, and molten pool behavior of the Ag7.5Cu/Cu10Sn (A/C) and Cu10Sn/Ag7.5Cu (C/A) interfaces were studied to reveal the influence of different building strategies. At the A/C interface, pre-printed Ag7.5Cu promoted Marangoni turbulence at a relatively low energy density (E_A/C_ = 125 J/mm^3^). Due to the recoil pressure, the molten pool at the A/C interface transformed from a stable keyhole mode to an unstable keyhole mode. These phenomena promoted the extensive migration of elements, forming a wider diffusion zone and reduced thermal cracking. At the C/A interface, the molten pool was rationed from the conduction mode with more pores to the transition mode with fewer defects due to the high energy density (E_C/A_ = 187.5 J/mm^3^). This work offers a theoretical reference for the fabrication of HETC multi-material structures via LPBF under similar conditions.

## 1. Introduction

The typical highly electrically and thermally conductive metals (HETCMs) (e.g., Ag, Au, Cu) are widely used in various fields because of the special characteristics [1]. Ag has been used in smart electronics [2], green energy [3], wearables [4], jewelry [5], medicine and health care [6], and other emerging fields due to its excellent electrical [4], thermal [7], reflective [8], and antibacterial [9] properties. Cu has high conductivity [10], heat conduction [11], corrosion resistance [12] and toughness [13], and is widely used in electric power [14], heat dissipation [15], aerospace [16], marine applications [17], and other fields. However, the relatively high price, low hardness, low yield strength, and poor wear resistance of Ag limit its further application [18]. While Cu is widely used in a pure and an alloy form due to its machinability and relatively low cost.

Metallic additive manufacturing (AM) is an emerging technology that can efficiently produce functionally graded materials (FGMs), resulting in improved performance through gradual changes in the composition or microstructure [19]. Multi-material structures are typical FGMs [20]. By integrating the structures and functions of multiple materials, the combined properties of the multiple materials (e.g., local wear resistance, high thermal conductivity, thermal insulation, chemical corrosion resistance, etc.) [21] can be achieved to meet the growing demands of the aerospace, biomedical, automotive, and jewelry industries, among others [22]. Laser powder bed fusion (LPBF) is a common metal additive manufacturing process [23,24] that has the advantages of precision manufacturing and multi-scale precision control [25]. In the LPBF process, the combination of laser rapid solidification and high thermal conductivity metal contributes to promote grain refinement [17], which provides the possibility to improve the comprehensive performance of HETCMs [1]. The smaller spots and thinner layers enable LPBF to fabricate metallic multi-material components with more complex structures and higher geometric resolutions [21], which are otherwise difficult or impossible to fabricate with the traditional manufacturing techniques [26] such as powder metallurgy [27], welding [28], casting [29], cold pressing [30], etc. Through LPBF technology, precious metal materials can be used efficiently, and its green and flexible manufacturing characteristics are also helpful in expanding the application of Ag materials in emerging high-tech fields.

Although the mentioned studies highlight the potential advantages of Ag and Cu, there are few articles related to the LPBF fabrication of Ag–Cu because of the expensive price of Ag and the difficulty of printing HETCMs [31,32]. For example, Robinson et al. [32] printed 99% pure Ag using single-tracks fabrication on Cu and steel substrates and found that the Cu substrates with high thermal conductivity and high reflectivity were not suitable for LPBF fabrication of pure Ag single-tracks. Xiong et al. [18] found that the grain refinement caused by the LPBF process contributed to the Vickers hardness of the LPBFed Ag alloy three times higher than that of the casting Ag alloy. John et al. [33] found that when using LPBF in situ alloying to fabricate Cu–Ag structures with different Ag contents, the pore number and average pore size of both the prefabricated and the annealed samples decreased with the increasing Ag content. Temel et al. [34] fabricated Ag-coated Cu cores through LPBF to obtain materials with higher conductivity and oxidation resistance at a relatively low cost. The above studies demonstrated the feasibility of fabricating Ag–Cu multi-material via LPBF. However, existing studies mainly use in situ alloying and pre-alloying. The fabrication of Ag–Cu multi-material LPBF using a sandwich structure has not been investigated.

Some LPBF-based metallic multi-material structures (MMS) have been investigated, such as copper–steel [20], copper–nickel [35], titanium–copper [36], aluminum–copper [37], etc. The research contents involve the interface characteristics of multiple materials, including the interfacial microstructure, the elemental diffusion mechanism, the interfacial melting mode, etc. Chen et al. [38] printed 316L–Cu10Sn MMS using LPBF and found that many dendritic cracks in the diffusion zone came from the different physical properties of 316L and Cu10Sn. Wei et al. [36] printed Cu10Sn and Ti6Al4V with different construction strategies using LPBF and found that the thin elements reaction zone at the interface contributed to the delamination of dissimilar materials, while more extensive element migration enhanced the bonding strength of multi-material interfaces. Tan et al. [39] found that due to the high cooling rate of the copper substrate, gradient/fine dendrite grains were generated at the interface of the LPBFed copper–steel MMS, resulting in an ultra-high copper–steel bonding strength. Liu et al. [20] investigated the correlation of energy density with the Marangoni convection-driven molten pool flow behavior in 316L–Cu10Sn multi-material LPBF fabrication.

Previous studies have shown that different building strategies in metallic multi-material LPBF fabrication affect interface behavior [40,41], but there is no related study on Ag–Cu multi-material. The multi-material used in the current research are mainly dissimilar materials with certain thermo-physical properties (e.g., thermal conductivity, thermal expansion coefficient, laser absorptivity, melting temperature, etc.), but the HETCMs MMS fabricated by LPBF has not been reported yet. Therefore, a study of the bonding characteristics of the interface between Ag alloy and Cu alloy is helpful in breaking down the barriers of HETCMs MMS additive manufacturing and expanding the application of HETCMs in various industries.

In this study, the Ag7.5Cu/Cu10Sn/Ag7.5Cu multi-material sandwich structures were printed by LPBF. The sandwich structure had two interfaces, the Ag7.5Cu/Cu10Sn interface (Cu10Sn was printed on Ag7.5Cu), and the Cu10Sn/Ag7.5Cu interface (Ag7.5Cu was printed on Cu10Sn). The interfacial characteristics (such as morphology, defect, microstructure, and element diffusion), interfacial melting mode, and their formation mechanisms were investigated.

## 2. Methods and Experiments

### 2.1. Experimental Materials

**Figure 1:**
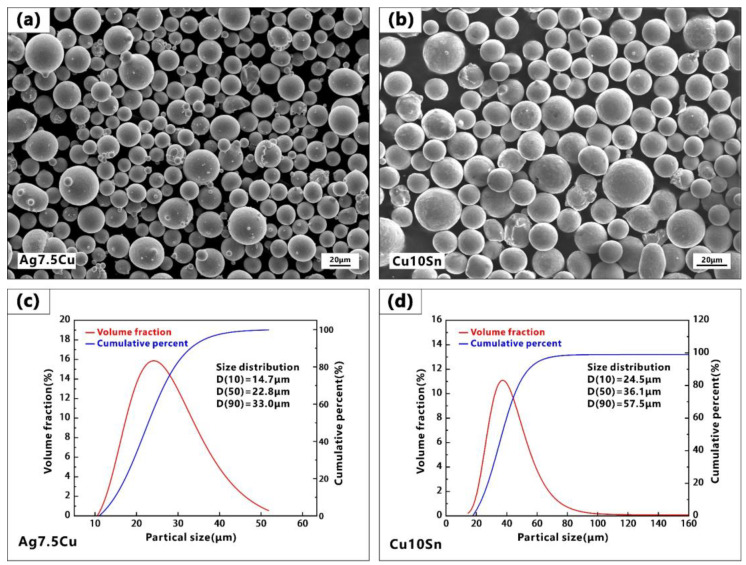
(**a**,**b**) SEM images of Ag7.5Cu and Cu10Sn powders, respectively; particle size distribution of (**c**) Ag7.5Cu powders and (**d**) Cu10Sn powders.

The spherical gas-atomized powders used in the experiment included Ag7.5Cu alloy powders (Figure 1a) and Cu10Sn alloy powders (Figure 1b), supplied by the Legor Group. Previous studies have shown that different powder particle sizes not only increased packing density but also improved the powder absorptivity of highly reflective metal powders through multiple reflections [42,43]. The particle size distribution of the powders was measured by a Mastersizer 2000 laser particle size analyzer. The Ag7.5Cu powders had a D10 of 14.7μm, a D50 of 22.8 μm, and a D90 of 33.0 μm, and the Cu10Sn powders had a D10 of 24.5 μm, a D50 of 36.1 μm, and a D90 of 57.5 μm, as shown in Figure 1c and 1d, respectively. The oxygen level of the powders was measured by a Leco ONH836 Oxygen/Nitrogen/Hydrogen elemental analyzer. The chemical compositions of two powders are shown in Table 1.

### 2.2. LPBF Process of Ag7.5Cu–Cu10Sn Multi-Material Structure

**Figure 2:**
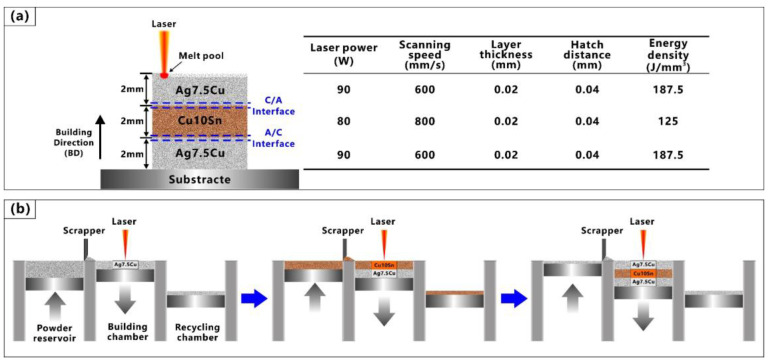
Schematic illustration of LPBF process for fabricating Ag7.5Cu/Cu10Sn/Ag7.5Cu multi-material samples: (**a**) building strategy and parameters; (**b**) printing process.

In this work, the sample was produced by LPBF, using a SISMA MYSINT100 system and an Nd: YAG fiber laser with wavelength of 1.06 μm, high power (up to 200 W), and small focus (30 μm). The sample was built in a nitrogen environment (residual oxygen content of 0.5 vol%). A schematic illustration of the multi-material LPBF process as shown in Figure 2. The sandwich structure sample was a 6mm x 6mm x 6mm cube consisting of Ag7.5Cu alloy on the bottom and top, and Cu10Sn alloy in the middle, with a 2 mm height of each metal (see Figure 2a). Due to the sandwich structure, two interfaces with different printing strategies could be observed in the same sample. The Ag7.5Cu/Cu10Sn interface and the Cu10Sn/Ag7.5Cu interface were referred to as the “A/C interface” and “C/A interface”, respectively. 

Differences in the melting point and thermal conductivity of different materials would lead to the insufficient melting of metals with higher melting points in the powder mixing region [19]. As metals with high reflectivity (Ag, Cu > 95%, 1064 nm) [44] and high thermal conductivity(Ag: 429 W/(m·K), Cu: 401 W/(m·K)) [45] require more laser energy to melt [23]. Based on our previous experiments [18], compared with Cu10Sn alloy, a higher energy density was used when printing the Ag7.5Cu alloy to avoid the insufficient melting of the Ag alloy. The optimized parameters for manufacturing the Ag7.5Cu alloy and Cu10Sn alloy were listed on the right side of Figure 2a, and a chessboard scanning strategy was applied to reduce the residual stress. The volume energy densities (*E_d_*) were calculated based on Equation (1) [46]. 

Figure 2b shows the LPBF printing strategies for fabricating the samples. After the first part of the Ag7.5Cu printing was completed, the Ag7.5Cu powders in the powder reservoir and powder recycling chamber were removed and cleaned, and then, the Cu10Sn powders were re-filled into the powder reservoir to continue the printing. This step was repeated until the entire multi-material sample was printed.

(1) $$E_{d}=\frac{P}{v\times h\times t}$$

where *v* is the scanning speed; *h* is the hatch distance; and *t* is the layer thickness.

### 2.3. Microscopic Feature

The side surface of the Ag7.5Cu/Cu10Sn/Ag7.5Cu sample was polished by the common metallographic method, up to 0.3 μm silica colloidal. The sample was polished mechanically to obtain mirror-like surface and etched with two different etchants. One etchant (50 mL ammonia water + 50 mL H_2_O_2_ (3 vol%) + 50 mL distilled water) was used for the Ag7.5Cu part. The Cu10Sn part was etched with 5 g FeCl_3_, 10 mL HCI, and 100 mL distilled H_2_O mixed solution. An optical microscope (OM) with a Leica M205 was used to characterize the surface morphology and defects. The interfacial microstructure and element distribution were observed and analyzed with a ZEISS Gemini 300 scanning electron microscope (SEM) equipped with an Smartedx energy dispersive spectroscope (EDS).

## 3. Results and Discussion

### 3.1. Interfacial Morphology and Defects

**Figure 3:**
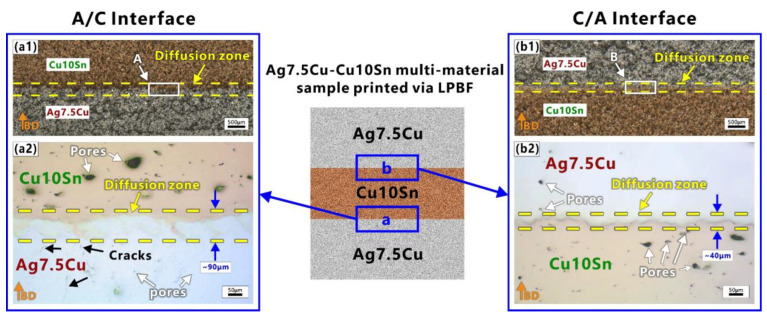
Optical images of two interfaces: (**a1**) macroscopic images of the A/C interface; (**a2**) enlarged view of area A in (**a1**); (**b1**) macroscopic images of C/A interface; (**b2**) enlarged view of area (B) in (**b1**). Orange arrows indicate the building direction (BD).

Figure 3 shows the optical mesoscopic images of the two interfacial regions of the Ag7.5Cu/Cu10Sn/Ag7.5Cu sandwich structure sample fabricated using LPBF. Three regions could be observed in the interface: the Ag7.5Cu region, the diffusion zone, and the Cu10Sn region. The diffusion zone of the A/C interface (~90 μm) was wider than that of the C/A interface (~40 μm), as indicated by the yellow dash lines in Figure 3(a1,a2) and Figure 3(b1,b2), respectively. No obvious mesoscopic defects (delamination or cracking) were observed in the binding regions (see Figure 3(a1,b1)), and the Ag7.5Cu was firmly metallurgically bonded to the Cu10Sn part. On the one hand, Ag and Cu were metallurgically compatible. As the Ag–Cu alloy system was an eutectic [47], the maximum solid solubilities of Cu in Ag at the eutectic temperature (779.1 °C) and at room temperature (20 °C) were 8.27 wt% and <1 wt%, respectively [47]. On the other hand, the large difference in the coefficient of thermal expansion (CTE) between dissimilar metals was one of the main causes of interfacial bonding defects [48,49], while the CTEs of Ag (19 ppm/K) and Cu (17 ppm/K) were relatively close [50], resulting in good metallurgical bonding in the LPBF manufacturing process.

**Figure 4:**
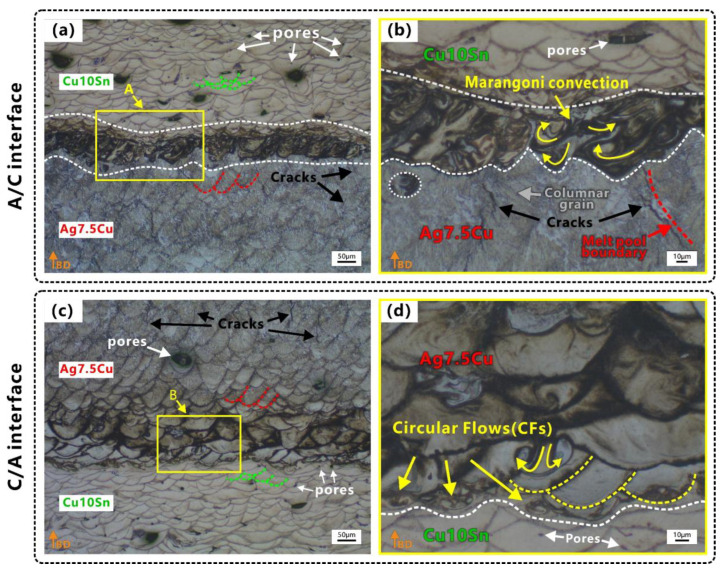
Characteristics of two interfaces after etching: (**a**) mesoscopic morphology at A/C interface; (**b**) enlarged view of area A in (**a**); (**c**) mesoscopic morphology at C/A interface; (**d**) enlarged view of area B in (**c**). Orange arrows indicate the building direction (BD).

Figure 4 shows the microstructure of the two interfaces after etching. The purpose of the etching was to better reveal the morphology of the molten pool. The overlapped molten pools with ‘fish scale’ [51] solidification lines could be clearly identified in the two interfaces, as shown in Figure 4. During LPBF fabrication, as new layers were deposited on top, a certain amount of the previously deposited layers were remelted [52,53], and the changes in the depth and shape of the molten pool resulted in irregular, wavy-shaped tracks [54]. Micro cracks and micro pores were observed in the Ag7.5Cu region and the Cu10Sn region respectively, while fewer defects were observed in the two diffusion zones of two interfaces, as shown in Figure 3 and Figure 4. Lack of fusion (LoF) [55] pores could be seen in the Cu10Sn region, mainly at the edge of the molten pool (see white arrows in Figure 4a,c). Microcracks were observed mainly in the Ag7.5Cu region (see black arrows in Figure 4a,c). Some cracks extended from the edge of the diffusion zone to the Ag7.5Cu side at the A/C interface (see black arrows in Figure 4b). These microcracks occurred at the molten pool boundary and extend in the same direction as the columnar crystal growth (parallel to the direction of the maximum thermal gradient). Microcracks were considered to be thermal cracks caused by high thermal stress due to high energy density, and they usually occurred and propagated along the grain boundaries [56]. The layer-by-layer melting and solidification characteristics of the LPBF process facilitated epitaxial growth, and it tended to induce columnar grains [57]. The columnar grains provided a long and straight path for crack propagation along the grain boundary [58], while the high solidification rate of Ag was not conducive to liquid backfill, making LPBFed Ag more prone to cracking. In addition, the unique solidification morphology caused by Marangoni convection was observed at the A/C interface, as shown in Figure 4b. In the LPBF process, the Marangoni convection, recoil pressure, and surface tension of the molten pool promoted elemental mixing and drove the molten pool into a keyhole mode [59,60]. Previous studies have shown that the large thermal stress induced by the high thermal gradients increased the possibility of crack generation [61]. Therefore, cracks were more likely to occur in the heat-affected zone (HAZ) below the keyhole mode molten pool [62]. This also explains why there were more microcracks below the diffusion zone of the A/C interface.

### 3.2. Element Diffusion

**Figure 5:**
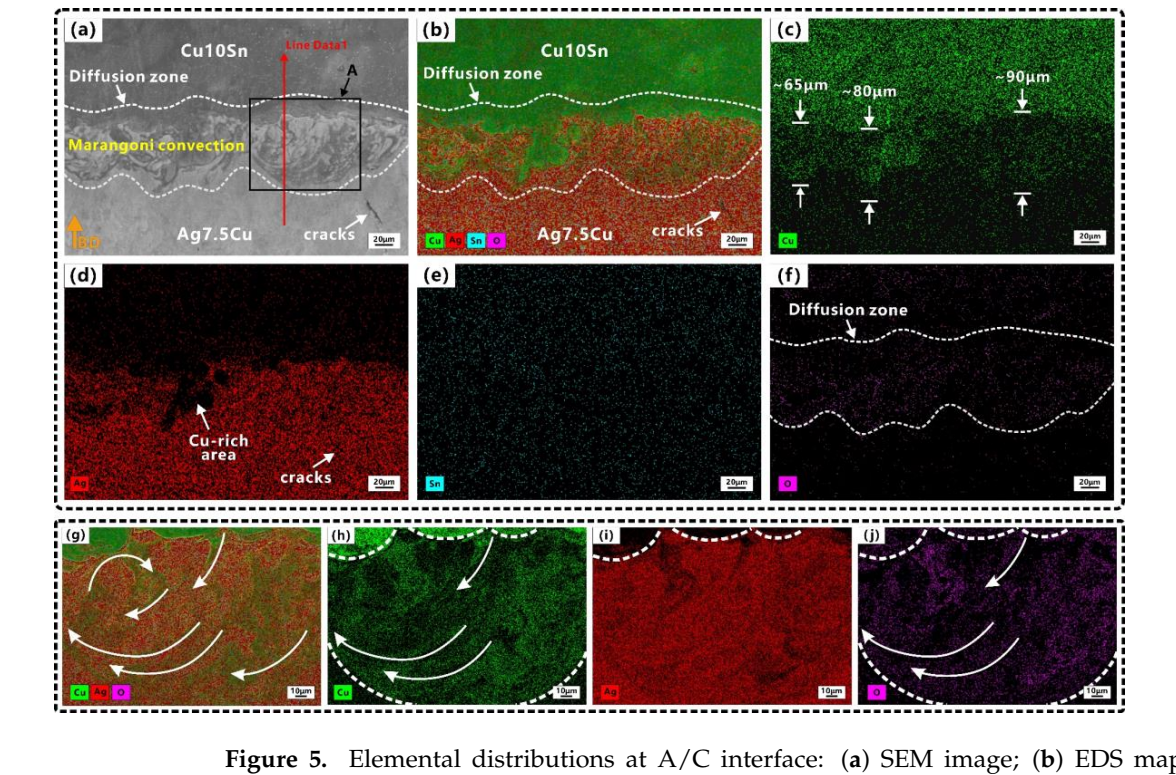
Elemental distributions at A/C interface: (**a**) SEM image; (**b**) EDS mapping of (**a**); (**c**–**f**) showing elemental distributions of Cu, Ag, Sn, O, respectively; (**g**) EDS mapping of area A in (**a**); (**h**–**j**) showing elemental distributions of Cu, Ag, O, respectively. Orange arrow indicates the building direction (BD).

**Figure 6:**
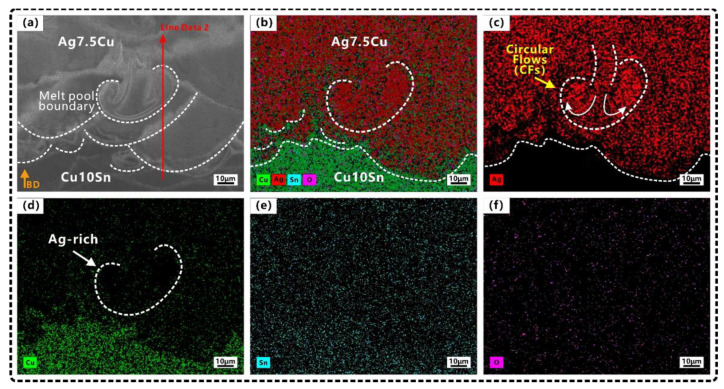
Elemental distributions at C/A interface: (**a**) SEM image; (**b**) EDS mapping of (**a**); (**c**–**f**) showing elemental distributions of Ag, Cu, Sn, O, respectively. Orange arrow indicates the building direction (BD).

**Figure 7:**
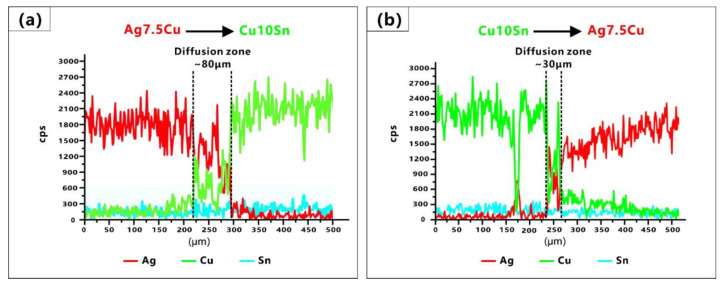
EDS line scan results along the scan path 1 (**a**) and 2 (**b**) presented in Figure 5a and Figure 6a, respectively.

The main element distributions of the A/C and C/A interfaces are shown in Figure 5 and Figure 6, respectively. Since both materials contained Cu elements, EDS mapping and line scan result (Figure 7) were combined for a comprehensive evaluation in order to better determine the width of the diffusion zone.

The mixing and dilution of Ag, Cu, and Sn were evident in the molten pool at two interfaces (Figure 5 and Figure 6). The element diffusion distance of A/C interface (65–90 μm) was wider than that of C/A interface (30–40 μm). Some strong Marangoni convection could be observed at the A/C interface (Figure 4b and Figure 5a), and circular flows (CFs) were observed at the C/A interface (Figure 4d and Figure 6c). Tan et al. [48] also found that when printing copper–steel multi-material, the high thermal conductivity of copper at the bottom resulted in a better interfacial bond strength. However, they only used one material with high thermal conductivity, and the other material was steel, which had poor thermal conductivity. During LPBF, the CFs in the molten pool were induced by Marangoni convection (ΔM), which was proportional to the surface tension (γ) and temperature gradient (ΔT) [63]. This phenomenon was enhanced by the underlying Ag/Cu because the high thermal conductivity of Ag/Cu increased ΔT, which enhanced the dynamics of the Marangoni convection of the molten pool and further facilitated element migration in the diffusion zone. This was especially the case at the A/C interface (underlying Ag) since the thermal conductivity of Ag was higher than that of Cu. This explains in one aspect why the stronger Marangoni convection and a wider diffusion zone were observed at the A/C interface.

Due to the complex non-equilibrium solidification process in the LPBF process [64], the long-term diffusion was limited during the rapid cooling process [65], which was not conducive to the generation of element portioning. Therefore, it did not have the conditions to produce large eutectic areas. Wang et al. [66] manufactured 925 Ag through LPBF and found that some submicron-sized eutectic structures were formed. The Ag–Cu phase diagram showed eutectic reaction at 28.1wt.% Cu [47]. The Cu-rich area (Figure 5d) was observed at the A/C interface. Figure 5g–j shows the mapping images of the molten pool of the area A in Figure 5a. The melting tracks with curved strips and swirls were clearly observed, which proved that Ag and Cu formed a good interpenetrating interface. The circular movement of the molten metal (Marangoni convection) in the molten pool contributed to the diffusion and homogenization of elements [67], which also promoted more eutectic formation in the diffusion zone. Due to the good liquidity and castability of eutectic alloys, thermal cracks were effectively reduced or even eliminated by increasing the amount of the eutectic phase [68]. The low melting eutectic structure backfilled the cracks and increased the grain boundary area to prevent crack propagation [69], and the alloy composition near the eutectic point of the phase diagram was also conducive to achieve high castability [70]. This also explains why there were fewer cracks in the diffusion zone.

Comparing Figure 5f and j with Figure 6f, the oxygen content in the strong Marangoni convection zone at the A/C interface was higher, and the mapping area of the oxygen content was consistent with that of Cu. Since the sample was constructed in a nitrogen atmosphere (0.5 vol% residual oxygen) and stored powders were used in the experiments. On the one hand, this phenomenon was attributed to the higher oxidation degree of Cu10Sn powders than that of Ag7.5Cu powders (see Table 1). On the other hand, powder oxidation may have had an effect on the Marangoni convection. As the shape of the molten pool was determined by the melting mode of the LPBF process, the different degrees of Marangoni convection at the A/C and C/A interfaces could lead to differences in the melting modes at the two interfaces. This will be discussed in Section 3.3.

### 3.3. Melting Mode in Multi-Material LPBF

The melting mode in the multi-material interfacial region led to different molten pool morphologies and significantly affected the distance of the element migration [51]. Therefore, different melting modes were very important for the interfacial bonding of multi-material.

In this study, the diffusion zone of the A/C interface (~80 μm) was wider than that of the C/A interface (~30 μm) (Figure 7). The migration distance of the elements at the A/C interface was about twice that at the C/A interface. The different width of the diffusion zone was due to the different melting modes in the manufacture of LPBF-fabricated multi-material. Qi et al. [59] believed that there were three modes in the LPBF fabrication of Al7050: the keyhole, the transition, and the conduction modes. The keyhole mode produced a deep and narrow V-shaped or goblet molten pool; the conduction mode produced a semicircular molten pool; and the transition mode was between the first two. These three modes could be identified by the ratio of molten pool depth (D) to width (W) (D/W, the aspect ratio of the molten pool cross-section), the keyhole mode (D/W ≥ 0.55), the conduction mode (D/W < 0.25), and the transition mode (0.25 ≤ D/W < 0.55). In comparison, Ag and Cu had higher reflectivity and thermal conductivity than Al, and the solidification rates of Ag and Cu were faster. Therefore, there would be differences in the formation mechanism of the Ag–Cu molten pool. It was necessary to make a comprehensive judgment based on the morphology of the molten pool, the ratio of molten pool depth to width (D/W), and the ratio of molten pool depth to layer thickness (D/L). The molten pool morphology gave a direct indication of the melting mode, while D/W could be used to assist in judging the transformation of the molting mode, and D/L indicated the penetration depth.

#### 3.3.1. Melting Mode at the A/C Interface

**Figure 8:**
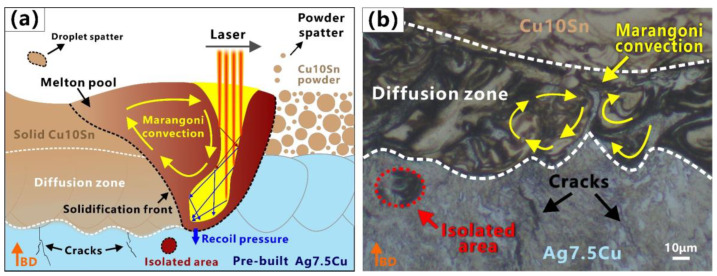
Melting mode of A/C interface in multi-material LPBF: (**a**) schematic illustration of molten pool behavior; (**b**) molten pool morphology at A/C interface. Orange arrows indicate the building direction (BD).

At the A/C interface, it could be observed that the molten pools of the Ag7.5Cu region had goblet or V shapes (see the red dash line in Figure 4a); the depth of the molten pool was about 62 μm; and the D/L was ~3.1 (deep penetration). Therefore, the melting mode of the Ag7.5Cu region belonged to the keyhole mode. When using a laser input p = 80 W, a scan speed v = 800 mm/s, and a volume energy density E_A/C_ = 125 J/mm^3^ (Figure 2a), strong Marangoni convection (Figure 4b) and strong element mixing (Figure 5b) were produced in the diffusion zone of the A/C interface. The depth of the keyhole would be further deepened when Cu10Sn was printed on Ag7.5Cu, since the laser beam reflected several times along the keyhole could significantly increase the laser absorption rate [71]. This contributed to the more sufficient melting of highly reflective metals and further promoted the intense mixing of materials, resulting in good metallurgical bonding at the A/C interface (see the schematic diagram in Figure 8). The molten pool subsequently transitioned from the stable keyhole mode (D/W~0.59) to the deeper and unstable keyhole mode (D/W~0.75, D/L~3.5). Due to the high cooling rate of the LPBF process (up to 10^6^–10^8^ K/s) and the high thermal conductivity of Ag, the solidification time was short. On the one hand, the instability of the critical keyhole caused by recoil pressure [72] released acoustic waves that pushed the keyhole tip away from the keyhole [73], which were trapped by the solidification front and formed an isolated area (Figure 8a,b). On the other hand, the strong recoil pressure promoted the deeper penetration of Cu10Sn by the Marangoni convection, which in turn resulted in more extensive element migration (~80 μm) (Figure 7a). In addition, during LPBF, increasing oxygen content changed the temperature coefficient of the surface tension from negative to positive, thus changing the Marangoni convection from an inward centrifugal flow to an outward cardiac flow [74]. The comparison of the oxygen content in the stronger (Figure 5f) and weaker (Figure 6f) Marangoni convection regions indicated that the higher oxygen content in the powder could intensify the Marangoni convection and promote more agitation in the molten pool.

#### 3.3.2. Melting Mode at the C/A Interface

**Figure 9:**
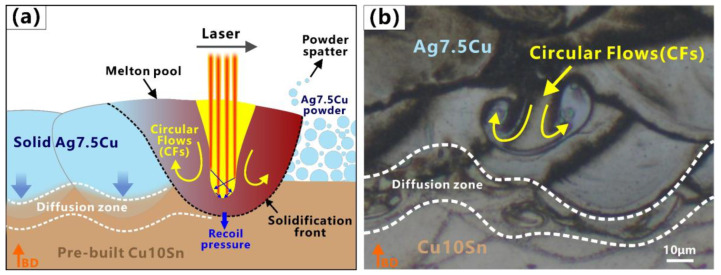
Melting mode of C/A interface in multi-material LPBF: (**a**) schematic illustration of molten pool behavior; (**b**) molten pool morphology at C/A interface. Orange arrows indicate the building direction (BD).

In the Cu10Sn region of the C/A interface, the D/W ratio of the molten pool was calculated to be ~0.41. According to Qi’s article, the molten mode of this proportion belonged to the transition mode. In this study, the molten pool in the Cu10Sn region had semicircular shapes (see green dash line in Figure 4c), the D/L was ~1.3 (thin penetration), and there was a presence of LoF. Therefore, we believed that the melting mode of the Cu10Sn region was more inclined to the conduction mode. In the diffusion zone of the C/A interface, higher laser power (p = 90 W), lower scanning speed (v = 600 mm/s), and higher volume energy density (E_C/A_ = 187.5 J/mm^3^) were used to print the Ag7.5Cu on the pre-printed Cu10Sn (Figure 2a). The molten pool transformed from the conduction mode to the transition mode (D/W ~0.44, D/L ~2), forming a deeper molten pool in the diffusion zone (see the yellow dash line in Figure 4d). In the transition mode, part of the recoil pressure was used to maintain the keyhole shape, while the other part pushed the molten pool in the opposite direction to the scanning direction [73], thus forming the CFs (see the schematic diagram in Figure 9a). Therefore, the shapes of molten pool in the diffusion zone were between the V shape and the semicircular shape, accompanied by a small range of CFs (see Figure 4d and Figure 9b). 

**Figure 10:**
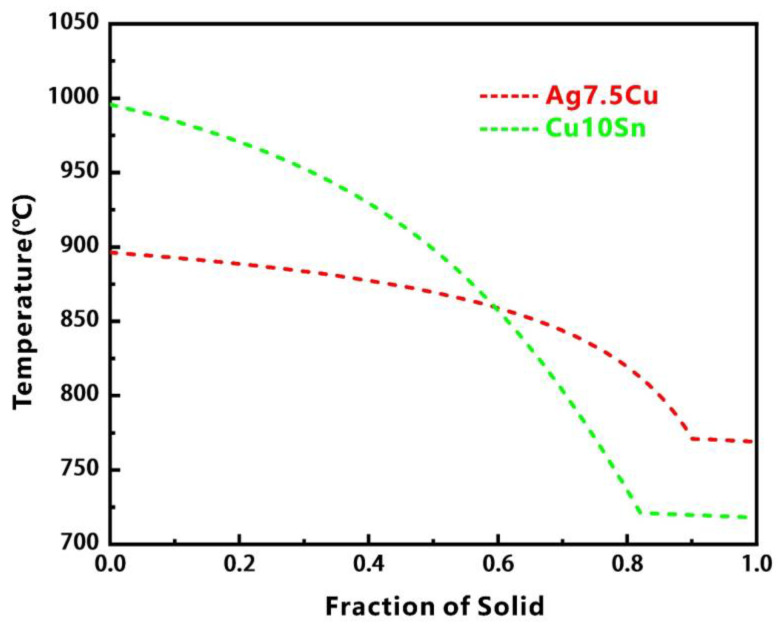
Scheil–Gulliver solidification curves of Ag7.5Cu and Cu10Sn.

During the LPBF process, when the powder bed absorbed sufficient laser energy, the previously solidified layer would remelt due to heat conduction. The longer the liquid time of the molten pool, the longer the diffusion time of the elements. Simulated Scheil–Gulliver solidification curves (Figure 10) showed that Ag7.5Cu had a narrower solidification temperature range than Cu10Sn. Due to the high solidification rate of Ag, the diffusion time was short, and the molten pool had already solidified rapidly before Ag was evenly distributed in the diffusion zone (see the Ag-rich zone in Figure 6d), resulting in the nonuniform and narrower diffusion zone (~30 μm) (see Figure 7b). Furthermore, when printing the upper layer of Ag7.5Cu, the pre-solidified diffusion zone could be covered, and some existing features such as CFs and defects (holes and cracks) could even be covered since remelting could repaired some defects [62]. This is one of the reasons why few CFs and defects were observed in the diffusion zone at the C/A interface. 

In summary, on the one hand, Marangoni turbulence was promoted when metals with high reflectivity were used as substrates. Marangoni convection facilitated eutectic formation in the diffusion zone, which could mitigate the crack defects. The multiple laser reflections inside the keyhole increased the laser absorption of the highly reflective metals. Marangoni convection and the recoil pressure facilitated the transition of the molten pool from the stable keyhole mode to the deeper and unstable keyhole mode. These promoted more extensive migration of the elements, resulting in a wider diffusion zone, and enhanced the metallurgical bonding at the A/C interface. Furthermore, the higher oxygen content in the Cu alloy powders affected Marangoni convection, resulting in more intense agitation in the molten pool and enhanced Ag–Cu interpenetration. On the other hand, using a higher energy density to print the C/A interface, CFs and recoil pressure changed the molten pool from the conduction mode to the transition mode and reduced defects in the diffusion zone. Due to the high cooling rate of LPBF and the rapid solidification rate of Ag, the diffusion time of elements was short. When the upper layer of Ag7.5Cu was printed, CFs and pore defects in the underlying diffusion zone were covered and reduced, respectively. These resulted in a narrower diffusion zone at the C/A interface than that at the A/C interface. 

## 4. Conclusions

In this study, the Ag–Cu multi-material structures were fabricated via LPBF. The characteristics and micromorphology (molten pool morphology, defects, element diffusion, etc.) of the A/C (printing Cu10Sn on Ag7.5Cu) and C/A interfaces (printing Ag7.5Cu on Cu10Sn) were investigated. The different melting modes and formation mechanisms of the two interfaces were discussed.

The following is a summary of the main conclusions:

(1) **The Ag7.5Cu/Cu10Sn/Ag7.5Cu sandwich structure was proposed and successfully printed using LPBF for the first time.** It was successfully printed with no obvious mesoscopic defects in the binding regions. Ag7.5Cu and Cu10Sn were firmly metallurgically bonded to each other. Some thermal cracks and LoF pores were observed in the Ag7.5Cu region and the Cu10Sn region, respectively, but fewer microscopic defects were found in the diffusion zone.

(2) **The high thermal conductivity of the substrate enhanced the Marangoni convection, which strengthened interfacial bonding strength and reduced the defects.** The diffusion zone of the A/C interface (~80 μm) was wider than that of the C/A interface (~30 μm). The strong Marangoni convection was observed at the A/C interface; it promoted an intense mixing of elements, resulting in more extensive elemental migration and a wider diffusion zone. The energy density used in the diffusion zone at the A/C interface (E_A/C_ = 125 J/mm^3^) was lower than that at the C/A interface (E_C/A_ = 187.5 J/mm^3^), indicating that the material with high thermal conductivity as a substrate could significantly promote Marangoni convection. The strong convection of the molten pool increased the amount of eutectic formation in the diffusion zone, thus mitigating the crack defects. Furthermore, the higher oxygen content in the Cu10Sn powders resulted in a stronger Marangoni convection in the powder mixing zone, enhancing the Ag–Cu interpenetration.

(3) **Defining the melting mode in the Ag–Cu interface region.** At the A/C interface, the keyhole mode was further enhanced by the Marangoni convection and the recoil pressure. The stable keyhole mode (D/W ~0.59, D/L ~3.1) enhanced the laser energy absorption and provided a large penetration depth. The unstable keyhole mode (D/W ~ 0.75, D/L ~ 3.5) was accompanied by more turbulent molten pools, which promoted elemental migration and resulted in better interfacial bonding strength. At the C/A interface, the molten pool transformed from the conduction mode (D/W~0.41, D/L~1.3) to the transition mode (D/W ~0.44, D/L ~2) at a higher energy density, reducing the defects. The underlying diffusion zone was covered by the upper Ag7.5Cu layer, resulting in a narrower diffusion zone and reduced the interfacial bonding strength.

## Figures and Tables

**Table 1:** Chemical compositions of Ag7.5Cu and Cu10Sn alloy powders (wt.%).

Materials	Ag	Cu	Sn	O	Others
Ag7.5Cu	Bal.	7.56	—	0.016	Less than 0.05
Cu10Sn	—	Bal.	10.20	0.038	Less than 0.05

## Data Availability

The data that support the findings of this study are available from the corresponding author upon reasonable request.
